# Workplace-based assessment of communication skills: A pilot project addressing feasibility, acceptance and reliability

**DOI:** 10.3205/zma001069

**Published:** 2016-11-15

**Authors:** Simone Weyers, Iman Jemi, André Karger, Bianca Raski, Thomas Rotthoff, Michael Pentzek, Achim Mortsiefer

**Affiliations:** 1 Heinrich-Heine-Universität Düsseldorf, Universitätsklinikum Düsseldorf - Centre for Health and Society (CHS), Institut für Medizinische Soziologie, Düsseldorf, Germany; 2 Johannes Gutenberg-Universität Mainz, Mainz, Germany; 3 Heinrich-Heine-Universität Düsseldorf, Universitätsklinikum Düsseldorf, Klinisches Institut für Psychosomatische Medizin und Psychotherapie, Düsseldorf, Germany; 4 Heinrich-Heine-Universität Düsseldorf, Universitätsklinikum Düsseldorf, Studiendekanat, Düsseldorf, Germany; 5 Heinrich-Heine-Universität Düsseldorf, Universitätsklinikum Düsseldorf, Klinik für Endokrinologie und Diabetologie, Düsseldorf, Germany; 6 Heinrich-Heine-Universität Düsseldorf, Universitätsklinikum Düsseldorf, Institut für Allgemeinmedizin, Düsseldorf, Germany

**Keywords:** Communication, competency, workplace based assessment, formative assessment

## Abstract

**Background:** Imparting communication skills has been given great importance in medical curricula. In addition to standardized assessments, students should communicate with real patients in actual clinical situations during workplace-based assessments and receive structured feedback on their performance. The aim of this project was to pilot a formative testing method for workplace-based assessment. Our investigation centered in particular on whether or not physicians view the method as feasible and how high acceptance is among students. In addition, we assessed the reliability of the method.

**Method: **As part of the project, 16 students held two consultations each with chronically ill patients at the medical practice where they were completing GP training. These consultations were video-recorded. The trained mentoring physician rated the student’s performance and provided feedback immediately following the consultations using the Berlin Global Rating scale (BGR). Two impartial, trained raters also evaluated the videos using BGR. For qualitative and quantitative analysis, information on how physicians and students viewed feasibility and their levels of acceptance was collected in written form in a partially standardized manner. To test for reliability, the test-retest reliability was calculated for both of the overall evaluations given by each rater. The inter-rater reliability was determined for the three evaluations of each individual consultation.

**Results:** The formative assessment method was rated positively by both physicians and students. It is relatively easy to integrate into daily routines. Its significant value lies in the personal, structured and recurring feedback. The two overall scores for each patient consultation given by the two impartial raters correlate moderately. The degree of uniformity among the three raters in respect to the individual consultations is low.

**Discussion: **Within the scope of this pilot project, only a small sample of physicians and students could be surveyed to a limited extent. There are indications that the assessment can be improved by integrating more information on medical context and student self-assessments. Despite the current limitations regarding test criteria, it is clear that workplace-based assessment of communication skills in the clinical setting is a valuable addition to the communication curricula of medical schools.

## Zusammenfassung

**Hintergrund: **Die Vermittlung kommunikativer Kompetenzen hat eine größere Bedeutung in den Curricula der Humanmedizin bekommen. Ergänzend zu standardisierten Prüfungen sollten Studierende im Rahmen arbeitsplatzbasierter Prüfungen in realen klinischen Situationen mit richtigen Patienten kommunizieren und ein strukturiertes Feedback bekommen. Ziel des Projektes war, eine solche formative Prüfmethode zum arbeitsplatzbasierten Assessment zu pilotieren. Von besonderem Interesse war zu ermitteln, ob Ärzt/innen die Methode für praktikabel halten und inwiefern die Studierenden sie akzeptieren. Darüber hinaus sollte die Reliabilität der Methode eingeschätzt werden.

**Methode: **Hierzu führten 16 Studierende in ihren hausärztlichen Lehrpraxen je zwei Beratungsgespräche mit chronisch kranken Patienten und zeichneten diese per Video auf. Bewertung und Feedback erfolgten jeweils unmittelbar nach dem Gespräch durch den geschulten Lehrarzt anhand des Berlin Global Rating (BGR). Ferner erfolgte eine Bewertung der Gespräche mit dem BGR durch zwei externe geschulte Prüfer anhand der Videos. Die Angaben der Ärzte und Studierenden zur Praktikabilität und Akzeptanz wurden schriftlich und teilstandardisiert erhoben und quantitativ sowie qualitativ ausgewertet. Zur Prüfung der Reliabilität wurde die Retest-Reliabilität der beiden Gesamtbewertungen durch die jeweiligen Prüfer berechnet sowie die Inter-Rater-Reliabilität der drei Bewertungen aller Einzelgespräche.

**Ergebnisse: **Die formative Prüfmethode wird von den Ärzten und Studierenden als gut bewertet. Sie ist relativ gut in den Arbeitsalltag zu integrieren. Der besondere Mehrwert besteht im persönlichen, strukturierten und wiederholten Feedback. Die beiden Gesamtbewertungen des Patientengespräches durch den jeweiligen Prüfer korrelieren mäßig. Die Übereinstimmung der drei Prüfer hinsichtlich der Einzelgespräche ist gering.

**Diskussion:** Im Rahmen des Pilotprojektes konnte nur eine kleine Stichprobe von Ärzten und Studierenden in begrenztem Umfang befragt werden. Hierbei ergaben sich Hinweise zur Verbesserung der Prüfung durch die Integration von Kontextinformationen und studentischen Selbsteinschätzungen. Trotz der bislang noch vorhandenen Einschränkungen bezüglich der Testgüte ist deutlich geworden, dass die arbeitsplatzbasierte Bewertung kommunikativer Kompetenzen im klinischen Setting eine sinnvolle Ergänzung im Kommunikations-Curriculum einer medizinischen Fakultät ist.

## 1. Introduction

### 1.1 Background

In the international and German recommendations for designing curricula for medical degree programs, teaching communication skills is identified as one of the core objectives for successful medical education [1], [2], [3]. Programs have already been implemented at many German medical schools to teach and assess social and communicative competencies [4], [5], [6], [7]. At the University of Düsseldorf the interdisciplinary and longitudinal curriculum *CoMeD* has been put into place for teaching and testing communication skills. Assessment procedures have previously focused on basic and practical knowledge, as well as the application of what has been learned during an OSCE (Objective Structured Clinical Examination) using actors [8].

In addition, workplace-based learning at stations and GP training practices is seen as essential to the acquisition of communication skills and competencies [9], [10]. During clinical clerkships and practicums, physicians-to-be need to practice their skills communicating with patients in supervised surroundings. Students frequently feel they are left on their own and desire more active instruction and feedback from experienced physicians [11], [12]. In contrast to the often well-structured and supervised training sessions and seminars in the Skills Lab and the theoretical subjects [13], the learning process that takes place during a practicum is not adequately guided [14], [15]. Consequently, the question arises as to which formative assessment methods can be employed during the practicum to foster and structure the student learning process as it relates to communication.

The aim of a workplace-based assessment is to observe specific student performance during routine clinical work and to provide feedback to guide learning [16], [17], [18]. In medical education, structured observation and feedback in the workplace often concentrate on practical skills such as anamnesis or physical examination techniques. At present there are very few instruments available for differentiated workplace-based assessment of communicative competencies in complex doctor-patient interactions. Global ratings are more likely to be recommended for this purpose, as are methods for giving direct feedback to students [19], [20]. In this project the Berlin Global Rating (BGR) scale, which has been successfully used in summative simulation assessments [8], has been transferred to the practicum and drawn upon for the workplace-based, formative assessment of communication skills. To our knowledge, no prior experience with this has been gathered.

#### 1.2 Project aims

The goal of this pilot project was to test the possibilities and limits of a workplace-based assessment of doctor-patient communication using BGR that evaluates student performance in the clinical setting and provides structured feedback. Answers to the following questions were sought:

1. Is a formative workplace-based assessment using BGR feasible within the setting of a general medical practice?
2. What is the level of acceptance among students for this assessment method?
3. How reliable is BGR as a test instrument for the workplace-based assessment?

## 2. Methods

### 2.1 Project planning and recruitment

Representatives from the subjects covered by the communication curriculum participated in regular meetings concerning the design and development of the workplace-based assessment. Organization and realization took place over the course of a year through the efforts of a graduate assistant.

The resulting assessment was administered as part of the 14-day practicum in general practice during the fourth year of study for which students are prepared in case-based seminars on general medicine and training sessions on communication [7]. One of the main learning objectives is independently holding patient consultations. Students are supervised and guided by mentoring physicians who serve as medical student trainers in one-on-one teaching sessions. Students also have access to an online logbook [21].

First, only mentoring physicians with GBR training were recruited for the pilot project. All students who were doing their GP practicum at one of these practices were then invited to voluntarily participate in the project. Efforts were made to increase the response rate by holding informational meetings and approaching individuals personally. As a result, 16 students agreed to participate and, using a set of guidelines, were informed about the project’s aims and procedure. Student participation was rewarded with a 20 Euro gift certificate for books. The students were each assigned to one of nine medical practices. The participating nine mentoring physicians were then informed in detail a second time and given instructions based on the guidelines.

The mentoring physician selected and briefed the patients on-site at the practice. Patients were given written information explaining the study procedure before they gave their consent to participate and be recorded on video; participating patients also acknowledged the provisions for data privacy.

#### 2.2 Assessment of communicative competencies using the Berlin Global Rating Scale

The BGR scale developed by Hodges and McIlroy [22] and validated in German by Scheffer [23] was used as a test instrument for the workplace-based assessment. The BGR scale measures the quality of communication with four items: empathy, structuring doctor-patient conversations, verbal and nonverbal expression. Using a five-point Likert scale, student performance regarding these aspects is rated, with clear definition of the extremes (e.g. for empathy: “The student does not respond or responds inappropriately to obvious verbal and nonverbal cues or the needs of the patient”=1 point versus “The student consistently responds with understanding to verbal and nonverbal cues and the needs of the patient or responds in an appropriate manner”=5 points). Students can earn a maximum of 20 points.

#### 2.3 Data collection and analysis

Students were assigned the task of holding and recording two consultations at the practice with chronically ill patients (e.g. within the context of a disease management program or check-up). Recording devices were borrowed from the university; students were explicitly asked not to use cell phones to record. The mentoring physician observed and rated the student’s performance according to the four BGR items. Detailed feedback was then given at the end. As an aid, there was an information sheet with concrete recommendations for giving feedback on each of the items (see attachment 1 ).

After completion of the practicum, the video recordings were given to the project team so that consultations could be blindly evaluated by two independent raters. This task was also assumed by other persons who were familiar with BGR. As a result each consultation was evaluated by three different raters.

Finally, the nine mentoring physicians were surveyed using a partially standardized questionnaire regarding the feasibility of the assessment method in the context of routine clinical practice. Information was collected on sociodemographic profile, amount of time (four-point scale from “very time intensive” to needing “very little time”), and the overall evaluation of the assessment method (expressed as a standardized academic grade). In open-ended responses physicians were also able to comment on the reactions of the patients, practicability in terms of daily workloads, added value, transferability, and suggestions for improvement.

Students were asked in writing about their experiences and overall evaluation of the assessment method (also expressed as a standardized academic grade). In open-ended responses they were able to share what they considered positive about the assessment method, and what they thought was problematic.

#### 2.4 Analyses

The quantitative information contained in the physician surveys was scrutinized to ascertain the ease with which the assessment method could be incorporated into daily routines. The open-ended responses were paraphrased, parsed and condensed using summarizing content analysis. To determine the level of acceptance among students, the content of their open-ended responses was also evaluated by content analysis.

To verify the test-retest reliability, both overall evaluations of each rater were correlated using Pearson’s correlation coefficient (p<=0.05). The inter-rater reliability, meaning the correlation between the mentoring physician and the two impartial raters, who only viewed the video material, was calculated as an intra-class correlation (ICC) (type C, model with mixed two-way effects, 95% confidence interval; see attachment 2 ). Values greater than 0.75 indicate good reliability [24]. The quantitative analyses were performed with IBM SPSS Statistics 22.

## 3. Results

### 3.1 Project participants and data material

Of the mentoring physicians, three were female and six male. The mean age was 49.6 years with the mean length of professional experience in general practice amounting to 17.7 years.

In addition, 12 female and four male students participated in the pilot and were assigned among nine GP training practices. At the time of the project the students were in their eighth semester of medical study. With only one exception, each student recorded two consultations resulting in a total of 31 consultations for analysis. The results are presented in terms of the three main issues.

#### 3.2 Feasibility

**Practicability on a daily basis:** seven of nine physicians reported having no problems implementing the assessment method. Two physicians stated that it can be difficult to implement depending on the patients coming into the practice, for instance, if there is insufficient space available for the assessment, or if the mentoring physician does not have enough time due to emergencies. The amount of time needed was viewed as being neither great, nor particularly small (see Table 1 (Tab. 1)).

**Added value: **In response to the question about the added value of the assessment method, two respondents named the feedback, two respondents the self-observation and gain in self-knowledge, and one person identified the reward of empathetic communication techniques. One person stated that the OSCE to assess communicative competencies with actors standing in for patients was sufficient.

**Transferability:** Eight of nine physicians were of the opinion that the method can be transferred to other communicative contexts, for example, for anamnesis or setting therapy goals. One person viewed the transferability as challenging.

**Improvements:** In response to the question about what could be added to the evaluation criteria, one person suggested a comparison between the self-assessment and those by observers. Another person recommended giving more detailed information on the context for the consultation; a third person proposed that the medical expertise of the students also be evaluated.

On average, the mentoring physicians rated the assessment method with “good.”

#### 3.3 Acceptance

On average the students also rated the assessment method positively (see Table 1 (Tab. 1)). The feedback provided by the students on the positive and negative aspects of the workplace-based assessment yielded the following points:

##### Positive aspects

**Authenticity: **Students frequently praised the fact that during the workplace-based assessment they had spoken with actual patients in an authentic setting under real conditions. The doctor-patient exchange was viewed as being more authentic than the interchange with actors posing as patients, as takes place in the OSCE.

**Time: **Several students wrote that they found it good that they were able to take time for the doctor-patient consultation.

**Feedback: **Most students stated that they had received direct, personal and differentiated feedback from their mentoring physician. This feedback was given in part directly after the consultation and later as part of the joint analysis of the recorded material. One participant wrote that this was the first time he had experienced this during the clinical phase of his medical education. Some students had the impression that the physicians addressed their strengths and weaknesses in a differentiated manner. It was frequently reported that BGR was helpful in structuring the feedback according to different aspects. Students also reported that repeating the assessment was helpful and presented an opportunity to apply the criticism from the first consultation. Progress was subjectively noticeable based on the second round of feedback. One student stated that he also found the feedback given by the patient useful. This was not intended to be part of this assessment, but does suggest a possible variation of the assessment method.

One student was of the opinion that all students, regardless of any participation in the research project, should have the opportunity to experience such a workplace-based assessment.

**Figure 1:**
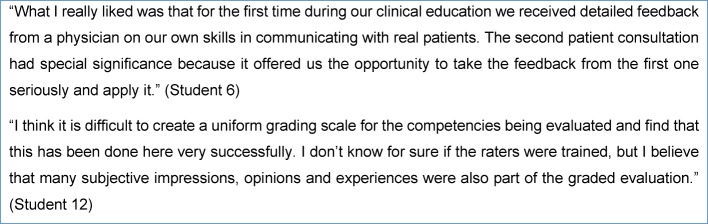
Selected quotes from students

Two particularly indicative statements by students are presented as quotations in the following Figure 1 (Fig. 1).

##### Negative aspects

**Setting: **One student found that it was impossible to plan for the consultation’s context, and that time was needed to adjust. Some students stated that the situation felt artificial. They felt inhibited by the running camera or the presence of the mentoring physician. One student reported that this also affected the patients.

**Assigned task:** Two students stated that the assignment was unclear. One felt a need for a more clearly formulated objective for the consultation; another found the physician was not clear on how to handle the videos.

**Role of the mentoring physician: **Some students reported that the assessment depended heavily on the teaching skills and commitment of the physician. They also expressed the concern that the evaluation was affected by the personal acquaintance between physician and student making it less objective.

**BGR:** On the topic of BGR one student responded that the scale was so finely graduated that it led to discrepancies between evaluations.

#### 3.4 Reliability

For the 31 patient consultations, the students earned a mean score of 15.55 out of a possible total of 20 points on the BGR scale. The two evaluations by the raters correlated with r=0.669, r=0.520, and r=0.653. The correlation between the mentoring physician and the two impartial raters was ICC=0.445. The correlation between pairs was ICC=0.619, ICC=0.301, and ICC=0.443 (see Table 2 (Tab. 2)).

## 4. Discussion

The aim of this project was to pilot a workplace-based assessment of communication skills at the University of Düsseldorf. The results are discussed here according to the three main issues.

### 4.1 Feasibility

The responses of the mentoring physicians indicate general concurrence. One very positive aspect is the near consensus among the physicians regarding the ease of integrating the evaluation and BGR feedback into the daily workloads shouldered in medical practices. No one indicated that the method demands “great amounts of time”, however, it is also true that no one stated that the method could be implemented with just a little effort here and there. A suitable point in time must be found, and it depends primarily on the presence of patients in the practice. Half of the nine physicians perceived added value in the new assessment method identifying, above all, the feedback. This corresponds not only with the philosophy underlying workplace-based assessments but also with statements made by the participating students. In this context, an interesting suggestion was made to include self-assessment on the evaluation sheet. Learners can give themselves feedback and compare their observations with the evaluations of other observers. Furthermore, the desire for more information on the reason for the consultation is understandable since the evaluation of the communication skills depends upon the context [25]. Suitable instruments could augment the formative assessment in valuable ways [26].

#### 4.2 Acceptance

Students consistently rated the assessment positively. Some criticism was expressed, but no one rejected the assessment method. As described in the literature [18], there is the impression that students rarely receive feedback on their clinical competencies. They particularly valued the chance to interact with “real” patients in “real” situations. A significant aspect of this workplace-based assessment is that students are given feedback immediately following their performance. In terms of the evaluation, some students felt that the BGR scale defined the grading criteria well and provided a structured and performance-centered evaluation, allowing the student to know their level of proficiency. Others, in contrast, felt that the mentoring physicians’ evaluations were too subjective and depended on how well they were trained or how long mentor and student had known each other. This is not an issue if the BGR is used for formative assessment. The opportunity of the second assessment to apply the criticism from the first round and potentially make progress was viewed as particularly positive.

According to Crooks [27], workplace-based assessment should be integrated into the learning process, feedback should be given immediately, students should have multiple chances to take assessments, and grading criteria should be clearly defined and formulated. The BGR scale can certainly serve as a framework here to organize some of the basic aspects of doctor-patient communication: structuring the medical consultation, responding to the patient, verbal and nonverbal communication. It clearly defines the expectations for each grade category and provides guidance for the feedback.

#### 4.3 Reliability

The results regarding the reliability of the assessment method are sobering. There is a moderate correlation among the overall evaluations of raters. This is to be expected with the short interval between the two consultations. In addition, there is a low level of correlation between the evaluations of the mentoring physicians and the impartial raters. From this it can be derived that it is urgently necessary to provide for physician training, joint standard setting, and clear definition of the context for consultations if this instrument is used for summative workplace-based assessment.

#### 4.4 Limitations

Despite multiple recruiting measures, only a small number of students could be acquired for participation in the pilot project. Consequently, the sample size is too small to draw any firm conclusions from the statistical results. The participants were not chosen randomly, but rather because of their interest and are not representative of the full student body. In terms of the participating medical practices, a selection bias must be assumed in favor of physicians who are generally open to the formative assessment method presented here. The students here involve a group that is interested in the topic of doctor-patient communication in a way that exceeds the average level of interest exhibited by medical students. However, a comparison with the OSCE scores shows that the student participants do not distinguish themselves significantly from the cohort average (results not shown here).

No validated survey instrument was available for the physician or student evaluations. The evaluation based on open-ended written responses is limited to a descriptive analysis of content; to evaluate more deeply held attitudes or motives, interviews or focus groups using qualitative research methods would be necessary.

#### 4.5 Conclusion

Despite various methodological limitations, there are indications for continued development of a workplace-based assessment of communicative competencies in the clinical setting. It has become clear that this formative assessment is a valuable addition to the communication curricula of medical schools. As part of the model study program at the University of Düsseldorf, the communication training program using actors to stand in as patients is now supplemented by a training program (with online logbook) using real patients that takes place during the clerkships of the fourth and fifth years of medical study and the 14-day practicum at a GP training practice [21]. The assessment presented here should continue to be tried out as a formative assessment method and developed further in response to evaluation results.

The results also demonstrate that use as a summative assessment requires a considerably greater amount of training and coordination than could be easily mastered by many medical practices. Together with other assessments, the concept discussed here involving observation and feedback yields a longitudinal portfolio of communicative competencies. It also poses a chance for and a challenge to medical schools to implement an improved culture of feedback in routine clinical practice.

## Acknowledgements

We wish to extend our gratitude to the QVM Kommission der Medizinischen Fakultät Düsseldorf for supporting this educational project. A special thanks goes to the students and general practitioners who participated in this project.

## Competing interests

The authors declare that they have no competing interests. 

## Informed Consent

Approval was given for this project by the Ethics Commission of the University of Düsseldorf’s Medical School (no. 7194).

## Supplementary Material

BGR with Feedback

ICC output

## Figures and Tables

**Table 1:**
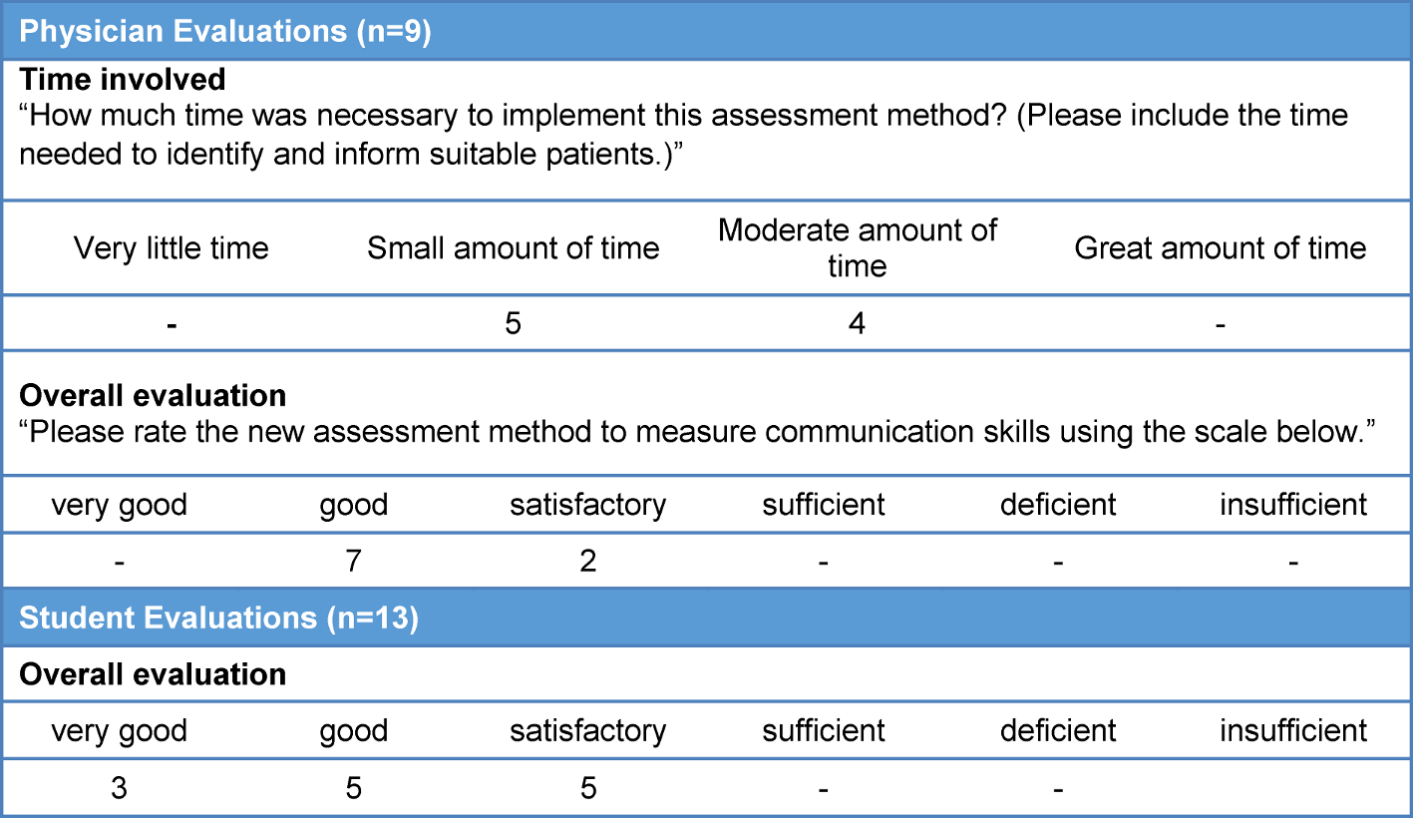
Feasibility and acceptance of the assessment method

**Table 2:**
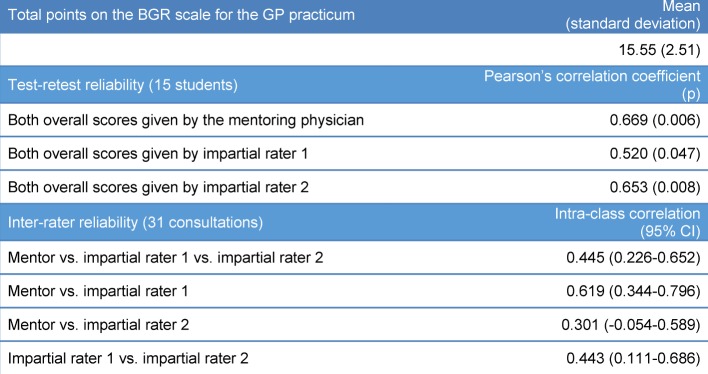
Reliability of the scoring; CI=confidence interval
