# Supplementary material for: Workplace-based assessment of communication skills: A pilot project addressing feasibility, acceptance and reliability
Source: GMS J Med Educ. 2016 Nov 15;33(5):Doc70. doi: 10.3205/zma001069 (PMC5135413; doi:10.3205/zma001069)
Supplement: BGR with Feedback [file JME-33-70-s-001.pdf]

CoMeD

Kommunikation
Medizinische Ausbildung
Düsseldorf ______________

Berliner Global Rating Scale

**GESAMTPUNKTZAHL:**	

CoMeD-Ü-OSCE mit Verhaltens-Feedback

» Rückmeldung zu positivem Verhalten
» Anbieten von Verbesserungsmöglichkeiten

Rückmeldungen an den Gesprächspartner bitte „kurz & knackig“ halten:
Was erscheint Ihnen besonders wichtig, lehr- und hilfreich zu sein?

I. EINGEHEN AUF DIE GEFÜHLE UND BEDÜRFNISSE DES PATIENTEN (Empathie)

Der Studierende geht durchgehend verständnisvoll auf die (verbalen und nonverbalen) Hinweise und Bedürfnisse des Patienten ein *oder* reagiert angemessen.	5 4 3 2 1	Der Studierende geht nicht auf offensichtliche (verbale und nonverbale) Hinweise und Bedürfnisse des Patienten ein *oder* reagiert unangemessen.

„Mir hat gut gefallen, dass Sie...
- ❒ auf verbalen / nonverbalen Gefühlausdruck eingegangen sind.
- ❒ Emotionen angesprochen haben.
- ❒ verständnisvoll reagiert haben.
- ❒ Belastungen anerkannt haben.
- ❒ Unterstützung und Trost angeboten haben.
- ❒ Beispiele: ______________________________

„Ich hätte mir gewünscht, dass Sie...
- ❒ verbalen / nonverbalen Gefühlausdruck wahrnehmen.
- ❒ Emotionen ansprechen.
- ❒ Ihr Verständnis für das Erleben des Patienten/in ausdrücken.
- ❒ die aktuelle Belastung des Patienten/in anerkennen.
- ❒ Unterstützung und Trost anbieten.
- ❒ Beispiele: ______________________________

II. LOGISCHER ZUSAMMENHANG DES GESPRÄCHS (Struktur)

Das Gespräch ist hervorragend organisiert: das Vorgehen zeigt, dass der Studierende in der Lage ist, das Gespräch zusammenhängend zu gestalten und die Gesprächsführung in der Hand zu behalten.	5 4 3 2 1	Das Gespräch ist nicht erkennbar organisiert: das Vorgehen wirkt zusammenhanglos oder der Patient muss den Verlauf des Gespräches festlegen.

„Mir hat gut gefallen, dass Sie...
- ❒ zu Beginn die Wahrnehmung/Wissen des Patienten erfragt haben.
- ❒ die Bereitschaft, die schlechte Nachricht zu erhalten, geklärt haben.
- ❒ gut strukturiert, zusammengefasst und Strategie erörtert haben.
- ❒ Beispiele: ______________________________

„Ich hätte mir gewünscht, dass Sie...
- ❒ zunächst den Kenntnisstand des Patienten explorieren.
- ❒ dessen Bereitschaft für schlechte Nachrichten erfragen.
- ❒ strukturiert vorgehen, zusammenfassen, Therapie erörtern.
- ❒ Beispiele: ______________________________

III. VERBALER AUSDRUCK

Der Studierende kommuniziert in einer Art und Weise, die es dem Patienten leicht macht, ihn zu verstehen *oder* er kommuniziert angemessen mit dem Patienten (z.B. hinsichtlich Wortwahl, Grammatik, Intonation, Lautstärke, Stimmmodulation, Sprechtempo und Aussprache).	5 4 3 2 1	Der Studierende kommuniziert in einer Art und Weise, die es dem Patienten erschwert oder unmöglich macht, ihn zu verstehen *oder* er kommuniziert unangemessen mit dem Patienten (z.B. hinsichtlich Wortwahl, Grammatik, Intonation, Lautstärke, Stimmmodulation, Sprechtempo und Aussprache).

„Mir hat gut gefallen, dass Sie...
- ❒ sehr verständlich kommuniziert haben haben.
- ❒ sich in Wortwahl / Lautstärke / Sprechtempo gut angepasst haben.
- ❒ Beispiele: ______________________________

„Ich hätte mir gewünscht, dass Sie...
- ❒ verständlicher kommunizieren.
- ❒ sich in Wortwahl / Lautstärke / Sprechtempo besser anpassen.
- ❒ Beispiele: ______________________________

IV. NONVERBALER AUSDRUCK

Der Studierende bezieht den Patienten durch nonverbalen Ausdruck durchgängig ein *oder* motiviert ihn zur Gesprächsbeteiligung (z.B. durch Augenkontakt, Mimik, Gestik, Körperhaltung und Einsatz von Pausen).	5 4 3 2 1	Der Studierende schafft es nicht, den Patienten durch nonverbalen Ausdruck einzubeziehen u. frustriert ihn *oder* bringt ihn gegen sich auf (z.B. durch Augenkontakt, Mimik, Gestik, Körperhaltung und Einsatz von Pausen).

„Mir hat gut gefallen, dass Sie...
- ❒ durchgehend Blickkontakt gehalten haben.
- ❒ in Mimik / Gestik / Körperhaltung beteiligt waren.
- ❒ angemessene Pausen zugelassen haben.
- ❒ Beispiele: ______________________________

„Ich hätte mir gewünscht, dass Sie...
- ❒ den Blickkontakt zum Patienten aufrechterhalten.
- ❒ sich stärker in Mimik / Gestik / Körperhaltung beteiligen.
- ❒ Raum für Pausen geben.
- ❒ Beispiele: ______________________________
