# Supplementary material for: Workplace-based assessment of communication skills: A pilot project addressing feasibility, acceptance and reliability
Source: GMS J Med Educ. 2016 Nov 15;33(5):Doc70. doi: 10.3205/zma001069 (PMC5135413; doi:10.3205/zma001069)
Supplement: ICC output [file JME-33-70-s-002.pdf]

```
RELIABILITY
  /VARIABLES=Erstrater_A_Ges Zweitrater_A_Ges Drittrater_A_Ges
  /SCALE('ALL VARIABLES') ALL
  /MODEL=ALPHA
  /ICC=MODEL(MIXED) TYPE(CONSISTENCY) CIN=95 TESTVAL=0.
```

Reliability

Scale: ALL VARIABLES

Case Processing Summary

		N	%
Cases	Valid	31	100,0
	Excluded[a]	0	,0
	Total	31	100,0

a. Listwise deletion based on all variables in the procedure.

Reliability Statistics

Cronbach's Alpha	N of Items
,707	3

Intraclass Correlation Coefficient

	Intraclass Correlation[b]	95% Confidence Interval		F Test with True Value 0			
		Lower Bound	Upper Bound	Value	df1	df2	Sig
Single Measures	,445[a]	,226	,652	3,410	30	60	,000
Average Measures	,707[c]	,468	,849	3,410	30	60	,000

Two-way mixed effects model where people effects are random and measures effects are fixed.

a. The estimator is the same, whether the interaction effect is present or not.

b. Type C intraclass correlation coefficients using a consistency definition. The between-measure variance is excluded from the denominator variance.

c. This estimate is computed assuming the interaction effect is absent, because it is not estimable otherwise.

```
RELIABILITY
  /VARIABLES=Erstrater_A_Ges zweitrater_A_Ges
  /SCALE('ALL VARIABLES') ALL
  /MODEL=ALPHA
  /ICC=MODEL(MIXED) TYPE(CONSISTENCY) CIN=95 TESTVAL=0.
```

Reliability

Scale: ALL VARIABLES

Case Processing Summary

		N	%
Cases	Valid	31	100,0
	Excluded[a]	0	,0
	Total	31	100,0

a. Listwise deletion based on all variables in the procedure.

Reliability Statistics

Cronbach's Alpha	N of Items
,765	2

Intraclass Correlation Coefficient

	Intraclass Correlation[b]	95% Confidence Interval		F Test with True Value 0			
		Lower Bound	Upper Bound	Value	df1	df2	Sig
Single Measures	,619[a]	,344	,796	4,246	30	30	,000
Average Measures	,765[c]	,512	,886	4,246	30	30	,000

Two-way mixed effects model where people effects are random and measures effects are fixed.

a. The estimator is the same, whether the interaction effect is present or not.

b. Type C intraclass correlation coefficients using a consistency definition. The between-measure variance is excluded from the denominator variance.

c. This estimate is computed assuming the interaction effect is absent, because it is not estimable otherwise.

```
RELIABILITY
  /VARIABLES=Erstrater_A_Ges Drittrater_A_Ges
  /SCALE('ALL VARIABLES') ALL
  /MODEL=ALPHA
  /ICC=MODEL(MIXED) TYPE(CONSISTENCY) CIN=95 TESTVAL=0.
```

Reliability

Scale: ALL VARIABLES

Case Processing Summary

		N	%
Cases	Valid	31	100,0
	Excluded[a]	0	,0
	Total	31	100,0

a. Listwise deletion based on all variables in the procedure.

Reliability Statistics

Cronbach's Alpha	N of Items
,463	2

Intraclass Correlation Coefficient

	Intraclass Correlation[b]	95% Confidence Interval		F Test with True Value 0			
		Lower Bound	Upper Bound	Value	df1	df2	Sig
Single Measures	,301[a]	-,054	,589	1,863	30	30	,047
Average Measures	,463[c]	-,113	,741	1,863	30	30	,047

Two-way mixed effects model where people effects are random and measures effects are fixed.

a. The estimator is the same, whether the interaction effect is present or not.

b. Type C intraclass correlation coefficients using a consistency definition. The between-measure variance is excluded from the denominator variance.

c. This estimate is computed assuming the interaction effect is absent, because it is not estimable otherwise.

```
RELIABILITY
  /VARIABLES=zweitrater_A_Ges Drittrater_A_Ges
  /SCALE('ALL VARIABLES') ALL
  /MODEL=ALPHA
  /ICC=MODEL(MIXED) TYPE(CONSISTENCY) CIN=95 TESTVAL=0.
```

Reliability

Scale: ALL VARIABLES

Case Processing Summary

		N	%
Cases	Valid	31	100,0
	Excluded[a]	0	,0
	Total	31	100,0

a. Listwise deletion based on all variables in the procedure.

Reliability Statistics

Cronbach's Alpha	N of Items
,614	2

Intraclass Correlation Coefficient

	Intraclass Correlation[b]	95% Confidence Interval		F Test with True Value 0			
		Lower Bound	Upper Bound	Value	df1	df2	Sig
Single Measures	,443[a]	,111	,686	2,591	30	30	,006
Average Measures	,614[c]	,200	,814	2,591	30	30	,006

Two-way mixed effects model where people effects are random and measures effects are fixed.

a. The estimator is the same, whether the interaction effect is present or not.

b. Type C intraclass correlation coefficients using a consistency definition. The between-measure variance is excluded from the denominator variance.

c. This estimate is computed assuming the interaction effect is absent, because it is not estimable otherwise.

```
GET
  FILE='C:\Users\sweyers\MME\Masterarbeit Von der Simulation\Daten\Datenmat
rixGesamt H1 und H2.sav'.
DATASET NAME DataSet2 WINDOW=FRONT.
```
